# Nonintrusive Power Load Decomposition Based on Adaptive Graph Convolutional Neural Network

**DOI:** 10.3390/s26102978

**Published:** 2026-05-09

**Authors:** Pinzhang Zhao, Jian Wei, Lihui Wang, Yajuan Qiu

**Affiliations:** 1 Jiangsu Institute of Metrology, Nanjing 210023, China; lemon_jsmi@163.com (P.Z.); 13645162540@163.com (J.W.); 2 Key Laboratory of Micro-Inertial Instrument and Advanced Navigation Technology, Ministry of Education, School of Instrument Science and Engineering, Southeast University, Nanjing 210096, China; qiuyj0829@163.com

**Keywords:** nonintrusive power load decomposition, adaptive adjacency matrix, graph convolutional neural network, feature input dimension

## Abstract

To fully exploit the correlation between the operating states of appliances, an adaptive graph convolutional neural network (AChebNet) for nonintrusive power load decomposition is proposed. An adaptive adjacency matrix is defined to characterize feature dependencies and uncover the hidden internal connectivity between features at different nodes in the graph model. This paper introduces the adaptive neighbor matrix to the Chebyshev Spectral CNN (ChebNet). By integrating a predefined neighbor matrix generated based on time intervals, we construct adaptive graph convolutions to better learn the graph structure and extract deeper hidden features. We explore the input dimensions of the model and select multiple relevant features based on the Spearman correlation coefficient to evaluate their impact on model performance. The proposed model outperformed ChebNet in experiments, achieving a 48.87% reduction in the mean absolute error (MAE) for the disaggregation of five appliances, and the mean power disaggregation accuracy improved from 87.39% to 92.74%. With multi-feature inputs, the model surpassed single-feature inputs, reducing the MAE by an additional 16.86% and increasing accuracy from 92.74% to 94.58%. Therefore, AChebNet can be effectively applied to reduce decomposition error and enhance overall accuracy in nonintrusive load decomposition.

## 1. Introduction

Nonintrusive Power Load Monitoring (NILM) is a convenient, cost-effective, and versatile monitoring approach [1], which decomposes load data monitored at the aggregate point of a power system to identify the operational states of each type or load within it [2]. Compared to traditional intrusive methods, such as installing individual meters for each appliance or placing sensors on each load to monitor energy consumption, NILM does not require entry into individual loads to establish or modify circuits. Instead, it analyzes aggregated load data collected from a central point in the system to decompose and identify the energy consumption patterns of each device. This approach offers low equipment and maintenance costs, reliability, safety, and ease of rapid deployment, providing distinct advantages in engineering applications [3].

Substantial research has been conducted on NILM. With advancements in computer hardware, traditional machine learning and deep learning methods have also been introduced. The Hidden Markov Model (HMM) is a landmark machine learning method applied in NILM, which has evolved into various forms to meet practical application needs [4]. K. Li et al. proposed a nonintrusive load monitoring algorithm based on a Factorial Hidden Markov Model (FHMM), using the Viterbi algorithm to decode the FHMM and identify the optimal sequence of hidden states so as to realize the accurate calculation of each device’s power consumption [5]. The Modified FHMM (MFHMM) framework for NILM improves appliance usage pattern estimation by considering dependencies between appliance operational states and differential states [6]. Iterative K-means clustering is employed to identify appliance operating states. These methods provide interpretable state-transition modeling and are effective for simple appliance-state estimation. However, they usually depend on strong prior assumptions about appliance operating states and often struggle when multiple appliances exhibit overlapping or highly similar power patterns. In addition, their performance may degrade in complex residential environments with strong temporal variation and nonlinear load interactions.

Deep learning methods improve NILM performance by enhancing nonlinear feature extraction and representation learning. Ren Wenlong et al. proposed a nonintrusive load decomposition method based on a deep sequence translation model, combining a sequence-to-sequence architecture with gated recurrent units to leverage the temporal scale information and signal amplitude characteristics of appliance operational patterns for load energy decomposition [7]. Huang et al. introduced Deep Convolutional Generative Adversarial Networks (DCGANs) to improve the classification accuracy of appliance states (on/off) by converting low-frequency power data into image format [8]. Compared to traditional machine learning algorithms, deep learning offers superior feature extraction capabilities, efficient model representation, and strong robustness, leading to significant advancements in the solution of NILM problems. In recent years, NILM research has further advanced toward more expressive sequence modeling architectures. For example, STNILM employs a Switch Transformer to model both short- and long-duration appliance behaviors [9], NILMFormer explicitly addresses the non-stationarity of smart-meter subsequences [10], and MAMBA2NILM combines SENet attention with Mamba2 to improve efficient long-sequence modeling [11]. These recent studies indicate that advanced sequence models have become an important trend in NILM. Nevertheless, most existing deep models process NILM data as regular sequences in Euclidean space and mainly focus on temporal mapping between aggregate and appliance-level signals. As a result, they often fail to explicitly characterize the hidden dependency relationships among different feature dimensions or among sampling points. Their performance may also deteriorate when the input features are limited or when appliances have similar operating signatures.

In data analysis, graph models are emerging tools that utilize spectral graph theory to map elements within a dataset to nodes in a graph, forming a feature graph structure. The degree of association between nodes is represented by edge weights, with higher weights indicating a stronger connection. This model has been widely applied in fields such as traffic flow prediction, action recognition, recommendation systems, and fault localization in power distribution networks [12,13,14,15]. However, the application of graph models in NILM is still in the exploratory stage. In NILM, there are two main approaches to applying graph models: graph signal processing (GSP) methods constructed through event detection and graph convolution methods. Reference proposed a nonintrusive load disaggregation method based on spectral graph theory, which established a graph structure using the differences between adjacent sampling points of the user’s total load sampling signal [16]. A global smoothness function of graph signals was employed to reconstruct the unknown portions of electrical appliance graph signals. Reference introduced the CFSFDP graph Laplacian algorithm in nonintrusive load monitoring, constructing a prior graph structure for household appliances and utilizing Tikhonov regularization to reconstruct power signals [17]. However, GSP-based approaches are highly dependent on the accuracy of event detection, and their performance may deteriorate when appliance switching events are ambiguous, overlapping, or difficult to identify in noisy environments. To overcome these limitations, graph convolution methods have gradually attracted attention in NILM. Peng Binggang et al. proposed a NILM method based on graph data modeling and graph representation learning, utilizing a Graph Convolutional Network (GCN) with a residual mechanism to extract features and enhance load decomposition accuracy through improved post-processing techniques [18]. However, it relies on predefined graph structures, which may not be sufficient to capture the dynamic dependency relationships among load features. Athanasoulias et al. combined the sequence-to-sequence method to convert aggregated signals into graph structures, utilizing a GCN for encoding, and employed a Transformer-based decoder to predict the power consumption of specific appliances, but this decoder introduces a relatively high computational cost and does not explicitly characterize the hidden correlations among multiple electrical features [19].

Despite recent progress, existing NILM models still do not fully address the joint modeling of temporal dependency and hidden feature correlation, while some methods also suffer from strong reliance on event detection or high computational complexity. These limitations indicate the necessity of developing a new graph-based framework with stronger adaptability and better efficiency. Accordingly, this study proposes a nonintrusive load decomposition model for residential electricity consumption based on an adaptive graph convolutional neural network.

The main contributions of this work are as follows. In this work, a hybrid graph construction strategy is developed for NILM by combining a predefined temporal adjacency matrix with a feature-driven adaptive adjacency matrix, and the resulting graph structure is embedded into the ChebNet framework. This design enables the model to simultaneously characterize temporal dependency and feature dependency in load sequences. The differences between multidimensional and unidimensional feature inputs are explored. The proposed model achieves a lower mean absolute error and higher decomposition accuracy on the publicly available AMPds2 dataset [20].

## 2. Selected Features

Active power is a commonly used key feature in the selection of single-load features for nonintrusive load decomposition. Highly correlated with power decomposition, it effectively reflects the operational states of various appliances, and its intuitive nature and high distinguishability facilitate the identification of devices with low sampling rates and high power loads. Therefore, to select active power as a feature is justified in single-load feature selection.

Research indicates limitations in relying solely on active power as a feature variable for load decomposition and identification. In particular, with low-power appliances with similar power characteristics or insufficient load data, the exclusive use of power features often fails to achieve precise identification. Therefore, multi-dimensional load feature selection strategies are adopted to enhance decomposition accuracy. In the process of multi-feature selection, the Spearman correlation coefficient is utilized to evaluate the correlations among features [21], which allows us to filter out features with higher correlation levels, which are then used as multi-feature data inputs. The Spearman correlation coefficient is defined as

(1) $$\rho =1-\frac{6\sum d_{i}^{2}}{n(n+1)(n-1)}$$

where n is the number of samples in each group, and $d_{i}$ is the rank differences between the variable samples of the two groups. The Spearman correlation coefficient has the range [−1, 1]. A positive correlation is indicated if ρ > 0, and a negative correlation if ρ < 0. A larger absolute value of ρ represents stronger correlation.

Five electrical characteristics—voltage (V), current (I), active power (P), reactive power (Q), and apparent power (S)—were selected. The Spearman correlation coefficient was used to analyze the relationship between the power consumption of five representative appliances in the AMPds2 dataset, namely lamps, air conditioners, dishwashers, refrigerators, and televisions. The results indicate that steady-state voltage has the weakest influence on these five household loads and thus can be considered an uncorrelated feature. Since active power is the most commonly used and representative feature in conventional NILM, it was also retained as the single-feature input baseline in this study. On this basis, the selected correlated features were further used to construct the multi-feature input setting for comparative evaluation.

## 3. Graph Convolutional Data Preprocessing

### 3.1. Load Decomposition Framework

In NILM, the application of GCNs primarily involves two core framework strategies: sequence-to-sequence (seq2seq) and sequence-to-point (seq2point). Sequence-to-sequence learning uses deep networks to map an input sequence (aggregate power data in NILM) to an output sequence (power data of individual devices) [22,23,24]. However, long sequential inputs and outputs in NILM lead to complex training computations, which are limited by issues such as GPU memory and vanishing RNN gradients. To address this challenge, the sliding window approach is typically used, where a long input sequence is divided into shorter sequences, and mappings are established between them. However, this method may lead to edge smoothing in the output signal, as each output element is predicted and averaged multiple times.

Focusing on the above problem, NILM uses sequence-to-point learning, building on the sequence-to-sequence framework combined with the sliding window method, establishing a mapping between a single load input subsequence within a window and the output signal at the midpoint of its timeframe, allowing the model to focus on the central moment characteristics of the window rather than dealing with more complex edge output predictions [25,26]. Based on this, sequence-to-point learning is chosen to construct the NILM framework. This approach realizes the mapping of the undirected graph data, consisting of load energy consumption sequences, to the target load power value at the corresponding point of the graph.

### 3.2. Sliding Window Method

Data collection is enhanced by segmenting the data with a sliding-window approach. Assume that the total energy consumption sequence captured is S={S(1),…,S(T)}, where *T* is the total number of sampling points. Let N denote the length of the sliding window, within which the initial sequence of energy consumption is $S_{1}=\{S(1),\ldots ,S(N)\}$. Subsequently, the sliding window is incrementally shifted forward by one sample point, yielding a total of T−N+1 sequences of energy consumption.

### 3.3. Constructing Graph Data

**Figure 1:**
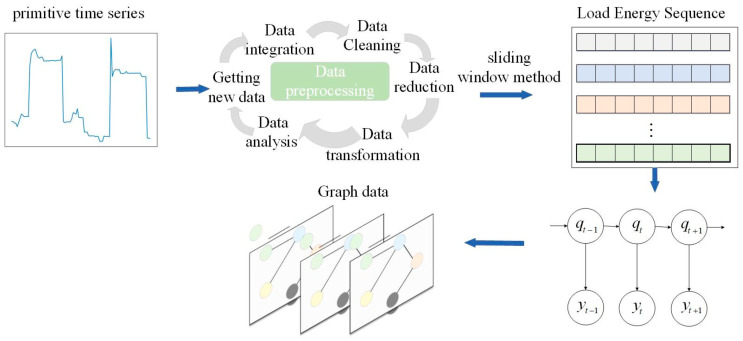
Schematic diagram of construction graph data.

In this study, each sliding-window load segment is modeled as an undirected weighted graph G = (V, E, A, X) where V is the node set, E is the edge set, A is the weighted adjacency matrix, and X is the node feature matrix. Each node represents one sampling point in the aggregate load sequence within the current window, and its feature vector contains the electrical measurements at that time instant. In the single-feature case, the node feature is the active power value; in the multi-feature case, the node feature vector consists of current, active power, reactive power, and apparent power. The edges connect different sampling points within the same window and describe their association relationships. To initialize the graph structure, the connections between nodes are predefined according to the temporal intervals between sampling points, so that temporally closer nodes are assigned larger edge weights and more distant nodes are assigned smaller edge weights. The weighted adjacency matrix in this stage is a predefined adjacency matrix. Since the graph is intended to characterize the structural dependency among sampling points rather than directional causality, it is constructed as an undirected graph. Therefore, the graph represents the internal structural relationship of the load sequence within a local time window. The process of constructing the graph data is illustrated in Figure 1.

The multiple segments of load consumption sequences obtained through sliding-window segmentation are used to independently construct undirected graphs for each segment based on the above process. The single-feature setting, using only active power, is retained as a baseline because active power is the most commonly used input in conventional NILM and allows the effectiveness of the proposed model itself to be fairly evaluated. The multi-feature setting is then introduced to further examine whether the selected correlated electrical features can provide additional complementary information and improve load disaggregation performance. When the node features of an undirected graph are composed of a single feature sequence, they are expressed solely by the active power value of the total load at that moment,

(2) $$X={\left[P_{1},P_{2},\ldots ,P_{N}\right]}^{T}$$

where N is the number of nodes, and nodes 1–N have respective active power values of $P_{1},P_{2},\ldots ,P_{N}$.

When the undirected graph is composed of multiple feature sequences, the node features of the graph are described collectively by the load current value, active power value, reactive power value, and apparent power value at that moment,

(3) $$X=\left[\begin{array}{cccc} I_{1} & P_{1}, & Q_{1}, & S_{1},\\ I_{2} & P_{2}, & Q_{2}, & S_{2},\\ \vdots & \vdots & \vdots & \vdots \\ I_{N} & P_{N}, & Q_{N}, & S_{N}, \end{array}\right]$$

For the definition of edge weights, regardless of how many feature sequences the undirected graph is composed of, weights based on time intervals are assigned [27]. The weighted adjacency matrix W is referred to as the predefined adjacency matrix, which is calculated by using a Gaussian kernel function in machine learning and can be expressed as

(4) $$W_{ij}=e^{-\frac{{\left\|t_{i}-t_{j}\right\|}^{2}}{2\sigma^{2}}}$$

where *t_i_* and *t_j_* denote the sampling instants corresponding to nodes i and j within the current sliding window, respectively, and |*t_i_*− *t_j_*| represents the temporal interval between the two nodes. σ is a scaling factor controlling the Gaussian decay of the edge weights. Due to differences in data dimensionality and other factors affecting the model’s convergence speed and training accuracy, node features are normalized to a range of 0–1.

### 3.4. Feature Normalization Processing

Preprocessing of the model input data is important to enhance the training speed, ensure efficient operation, and mitigate discrepancies in results caused by inherent data differences and dimensionality variations. In this paper, min-max normalization is used to normalize the data range to the interval [0,1]. The calculation formula is

(5) $$s_{i}^{\prime }=\frac{s_{i}-s_{\min}}{s_{\max}-s_{\min}}$$

where $S={\left[s_{1},s_{2},\ldots ,s_{i},\ldots ,s_{k}\right]}^{T}$, with respective minimum and maximum values $s_{\min}$ and $s_{\max}$. To ensure consistency and avoid data leakage, these two values are calculated from the entire training set rather than from each individual sliding window. Specifically, for each input feature, the global minimum and maximum values are first computed over all training samples and then fixed as normalization parameters. The same parameters are subsequently used for the validation set, test set, and inference stage. When processing multi-feature inputs, to ensure data consistency, min-max normalization is applied independently to each type of feature.

## 4. Adaptive Graph Convolutional Load Decomposition

### 4.1. Adaptive Neighborhood Matrix

In graph convolutional networks, the adaptive adjacency matrix can be implemented either through end-to-end learning using node embeddings [28] or by setting up a learnable mask matrix, whose parameters are continuously adjusted through training, allowing the adjacency matrix to adaptively modify itself [29]. In the process of graph modeling for nonintrusive load disaggregation, the formation of the graph structure must consider both temporal dependencies and the presence of feature dependencies. Hence, there may be hidden connections between energy consumption points, which similarly influence the information relationships among the nodes. Thus, we consider the input feature matrix as prior information and integrate several learnable parameters in an adaptive adjacency matrix that can represent feature dependencies,

(6) $$A^{adp}=V_{s}\cdot \sigma (W_{s}{\left(X^{\left(l-1\right)}\right)}^{T}+b_{s})$$

(7) $$A_{ij}^{adp^{\prime }}=\frac{\exp(A_{ij}^{adp})}{\sum_{j=1}^{N}\exp(A_{ij}^{adp})}$$

where *N* is the number of nodes; $X^{\left(l-1\right)}\in R^{N\times C_{l-1}}$ is the input to the *lth* adaptive graph convolution module; $V_{s}$, $b_{s}\in R^{N\times N}$, $W_{s}\in R^{N\times C_{l-1}}$ are learnable parameters; $C_{l-1}$ is the feature dimension of the input data in the *lth* layer; and σ(⋅) is a sigmoid activation function. $A^{adp^{\prime }}$ is dynamically computed based on the current inputs to the layer, and its elements semantically represent the strength of association between nodes i and j.

**Figure 2:**
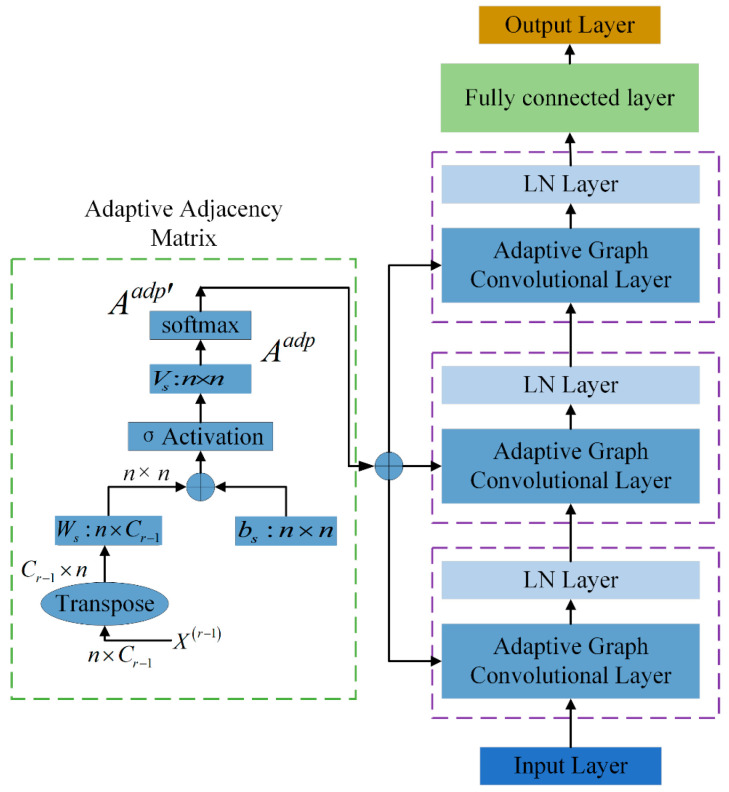
Structure of the adaptive adjacency matrix.

Figure 2 illustrates the structure of the adopted adaptive adjacency matrix. Our approach integrates the input feature matrix to facilitate the learning of the adaptive adjacency matrix, enabling a deeper understanding of the hidden intrinsic relationships between different node features. On the basis of the predefined adjacency matrix, an adaptive adjacency matrix is further learned automatically during training from the input feature matrix to capture hidden feature dependencies between nodes. Although adaptive graph construction has been explored in previous studies, the proposed method differs from existing graph-adaptive strategies in both design motivation and application context. Representative methods such as Graph WaveNet generally learn latent graph relations from trainable node embeddings or generic graph structure learning mechanisms, with the main objective of capturing hidden dependencies in general spatial–temporal graph data. In contrast, the proposed method is developed for NILM load decomposition and constructs the graph structure in a hybrid manner. Specifically, a predefined adjacency matrix is first established according to temporal intervals to preserve local temporal continuity, and a feature-driven adaptive adjacency matrix is then introduced to capture hidden dependency relationships among sampling points from the input electrical features.

### 4.2. ChebNet Graph Convolutional Network

The original spectral convolutional neural network has a global graph convolution kernel with a large number of parameters, resulting in high computational complexity [30]. ChebNet uses Chebyshev polynomial expansion to approximate graph convolution, which reduces computational complexity and preserves local connectivity by simplifying the parameterized frequency response function. ChebNet is expressed as

(8) L=D−A,

(9) $$\tilde{L}=\frac{2}{\lambda_{\max}}L-I_{N}$$

(10) X(l)=ReLU(∑k=0K−1θkTkL˜X(l−1))

where the *k*th term of a Chebyshev polynomial is recursively defined as $T_{k}\left(x\right)=2xT_{k-1}\left(x\right)-T_{k-2}\left(x\right)$; $T_{0}\left(x\right)=1$; $T_{1}\left(x\right)=x$; *L* is the Laplace matrix; A is the adjacency matrix; D is the degree matrix, $D_{ii}=\sum_{j}A_{ij}$; $I_{N}$ is the unit matrix, and *λ_max_* is the largest eigenvalue of the graph Laplacian matrix *L*; $\theta_{k}$ are learnable parameters; *k* represents a *k-order* neighbor; and the *lth* ChebNet module has respective output and input $X^{\left(l-1\right)}$ and $X^{\left(l\right)}$.

### 4.3. Adaptive Graphic Convolutional Networks

An adaptive adjacency matrix is introduced in ChebNet and is integrated with a predefined adjacency matrix based on time intervals to construct an adaptive graph convolutional layer. This allows the model to learn from a graph structure incorporating both temporal and feature dependencies, enabling deeper extraction of hidden internal features and achieving the mapping from energy consumption graph data to the target appliance power value at the midpoint of the graph. The adaptive map convolution layer is formulated as

(11) X(l)=ReLU(∑k=0K−1θkTkL˜⊙Aadp′X(l−1))

where ⊙ is the Hardman product, $X^{\left(l-1\right)}$ is the input to the *lth* adaptive graph convolution module, $X^{\left(l\right)}$ is the output of the *lth* adaptive map convolution module, $A^{adp^{\prime }}$ is the adaptive adjacency matrix dynamically computed from the current input, and $\theta_{k}$ is a learnable parameter.

### 4.4. Layer Normalization Layer

To enhance the network’s convergence speed and ability to generalize, we introduce layer normalization (LN) after each adaptive graph convolutional network layer, aiming to normalize the inputs to the neurons [31]. Layer normalization is expressed as

(12) $$X^{(l)\prime }=\frac{X^{\left(l\right)}-E[X^{\left(l\right)}]}{\sqrt{Var[X^{\left(l\right)}]+\varepsilon }}*\gamma +\beta$$

where $X^{{\left(l\right)}^{\prime }}$ is the result after normalizing $X^{\left(l\right)}$ by layer; $E[X^{\left(l\right)}]$ and $Var[X^{\left(l\right)}]$ are the respective mean and variance for $X^{\left(l\right)}$, ε takes the default value 1 × 10^5^, and γ and β are trainable parameters.

### 4.5. Graph Pooling Layer

To perform effective information fusion for all nodes in the graph, a one-time aggregation of the global information of the graph is achieved using a graph pooling layer, which is implemented by splicing the global average pooling and global maximum pooling [32].

(13) $$X=\frac{1}{N}\sum_{i=1}^{N}x_{i}∥\overset{N}{\underset{i=1}{\max}}x_{i},$$

where ∥ is a splice of the data by the same dimension. If the output feature dimension of the last graph convolution layer is F, the graph pooling layer generates a 2F-dimensional graph-level vector after concatenation.

### 4.6. Full Connectivity Layer

After preprocessing, adaptive graph convolution and graph pooling operations are performed on the original data. The deep features are mapped to the target appliance power using a fully connected layer, i.e.,

(14) $$X^{\left(l\right)}=\mathrm{ReLU}(W^{\left(l-1\right)}X^{\left(l-1\right)}+b^{\left(l-1\right)}),$$

where layer *l* has input $X^{\left(l-1\right)}$, output $X^{\left(l\right)}$, weight matrix $W^{\left(l-1\right)}$, and bias matrix $b^{\left(l-1\right)}$.

## 5. Model Structure Design

**Figure 3:**
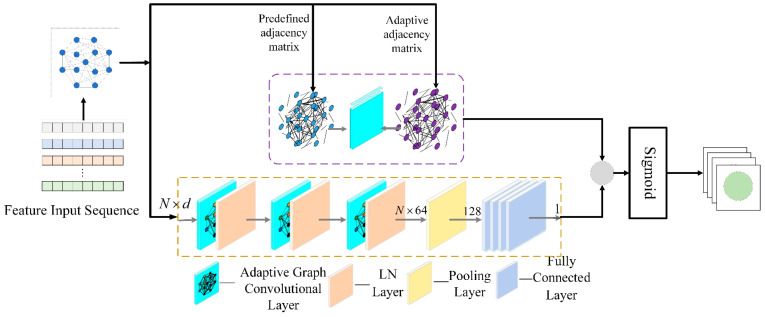
AChebNet model structure.

We propose a noninvasive load decomposition model based on an adaptive graph convolutional neural network, AChebNet, whose 11 layers include 3 adaptive graph convolutional layers, 3 normalization layers, 1 graph pooling layer, and 4 fully connected layers. The model draws on the sequence-to-point approach, where the network input is graph data derived from energy consumption sequences segmented by a sliding window [31]. The output corresponds to the power value of the target appliance at the midpoint of the sequence. Figure 3 shows the network structure.

The input to the network is graph data converted from energy consumption sequences segmented by a sliding window and preprocessed, such as by normalization. The input dimension of the first adaptive graph convolution layer corresponds to the data feature dimension, with an output dimension of 64. Both the second and third adaptive graph convolution layers have input and output dimensions of 64. After each of the three adaptive graph convolution layers, layer normalization is applied to achieve local response normalization. The graph pooling layer performs global average pooling and global max pooling on the output from the adaptive graph convolution module. The pooling results are concatenated along the same dimension to obtain the final pooled output, achieving a one-time aggregation of the graph’s global information. After extracting deep features through adaptive graph convolution and pooling operations, a four-layer fully connected network is used to map these features to the target appliance’s power. In this study, F = 64, so the pooled graph representation is 128-dimensional, which is then mapped to 64 neurons in the first fully connected layer. The subsequent fully connected layers contain 32, 16, and 1 neurons, respectively. Here, the output dimension of 1 represents a single predicted power value for one target appliance at the midpoint of the input window. For each appliance, an individual model is trained and tested separately.

A mean squared error loss function with L2 regularization is used to mitigate overfitting during model training. Samples are fed into the network in batches to improve training efficiency. The Adam optimizer is employed for backpropagation, adjusting network parameters until convergence. The best parameters during training are saved for testing. The main training hyperparameters are summarized in Table 1.

## 6. Experiments

### 6.1. Dataset Selection

Several public datasets have been widely used in NILM research. REDD is one of the earliest benchmark datasets for energy disaggregation research and has played an important role in promoting NILM studies; however, it contains data from a limited number of households and relatively short monitoring periods [33]. UK-DALE provides both appliance-level and whole-house measurements, and part of the dataset contains high-frequency mains data, which is beneficial for detailed load analysis; nevertheless, its number of monitored houses is still limited [34]. REFIT includes data from 20 UK households over a relatively long duration and is suitable for evaluating model generalization under realistic residential conditions, but its sampling granularity is lower and the available electrical feature dimensions are relatively limited [35]. In comparison, AMPds2 provides long-term residential monitoring data over two years and includes 11 electricity measurement attributes, together with appliance-level sub-metered data. These characteristics make AMPds2 more suitable for this study, since the proposed model aims to exploit multi-feature inputs and hidden dependencies among load features in a graph-based framework. Therefore, AMPds2 was selected as the main public dataset for model training and evaluation.

The effect of the proposed model was verified, using the AMPds2 public dataset for training and testing. An actual load data collection platform was designed and constructed to gather data and form a measurement dataset. The feasibility and generalization of AChebNet were further verified on a real test dataset. Taking into account the diversity of electrical appliances, lamps, heat-pump air conditioners, dishwashers, refrigerators, and televisions were selected as experimental objects for the AMPds2 dataset, and electric fans, televisions, and boiling pots for the measured dataset. For each target appliance, the samples obtained after sliding-window segmentation were independently divided into training, validation, and test sets in a ratio of 6:2:2. The validation set was used for model selection and hyperparameter tuning, whereas the test set was used only for the final performance evaluation. No appliance category was excluded from training or reserved entirely for testing.

### 6.2. Validation Analysis Based on Public Datasets

**Figure 4:**
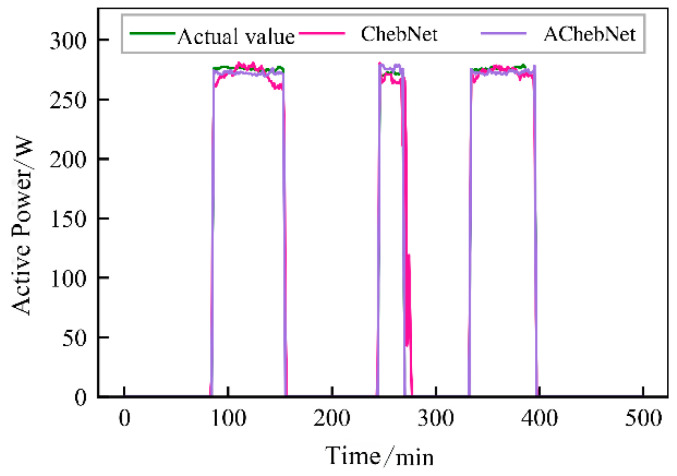
Decomposition effect of a lamp.

**Figure 5:**
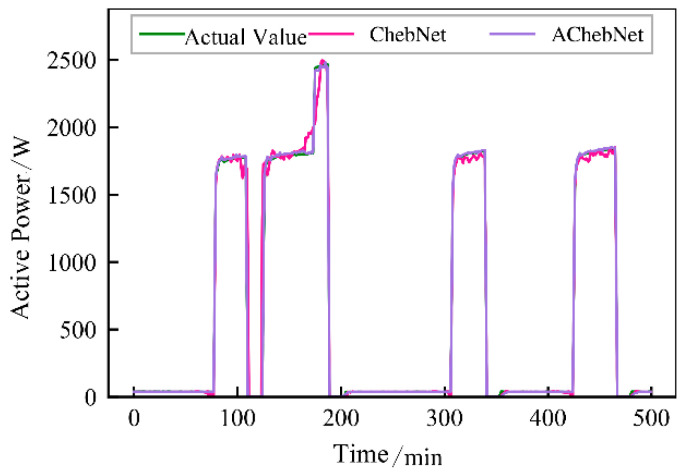
Decomposition effect of an air conditioner.

**Figure 6:**
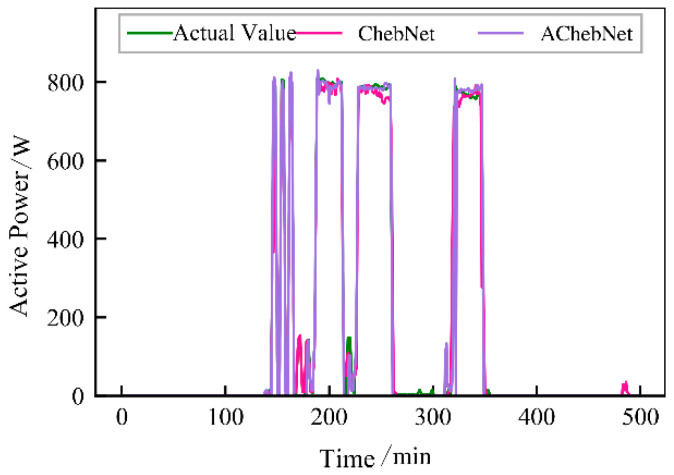
Decomposition effect of a dishwasher.

**Figure 7:**
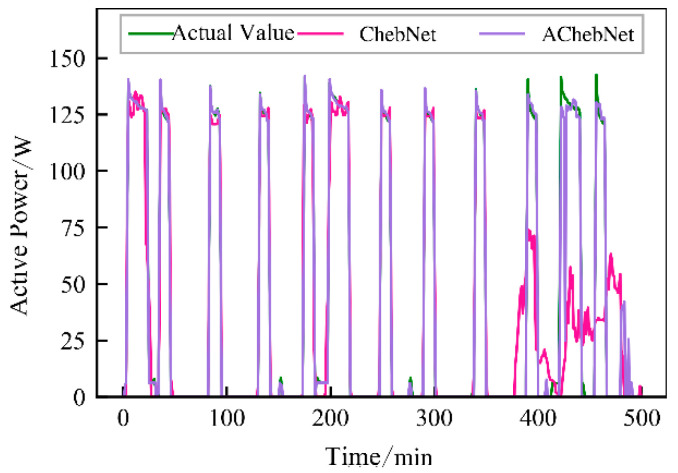
Decomposition effect of a refrigerator.

**Figure 8:**
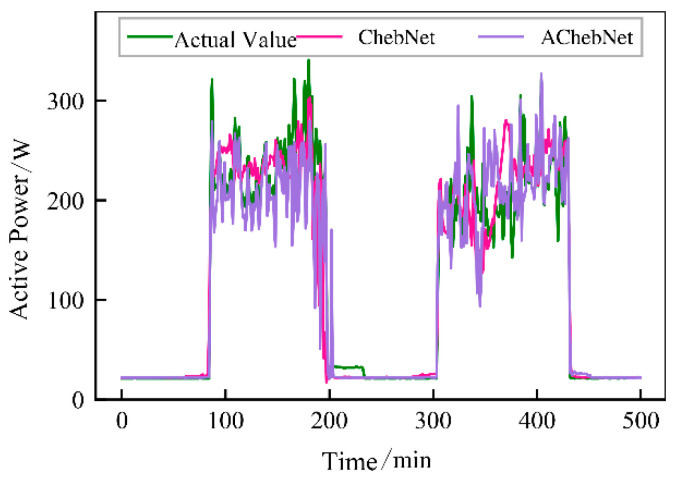
Decomposition effect of a television.

Active power electrical features were selected to validate the effectiveness of incorporating an adaptive adjacency matrix in the model. Simulations were conducted for both the ChebNet and AChebNet models, with the decomposition results for each appliance displayed in Figure 4, Figure 5, Figure 6, Figure 7 and Figure 8, which show that both ChebNet and AChebNet, which incorporates the adaptive adjacency matrix, achieve satisfactory decomposition results for each appliance. The models accurately detect the switching states of the appliances and effectively track power consumption. It is observed that for the decomposition of the five types of appliances, AChebNet consistently outperforms ChebNet, particularly in the case of refrigerators. This indicates that the incorporation of the adaptive adjacency matrix enhances decomposition performance for appliances.

Model performance was evaluated using the Mean Absolute Error (MAE), as used in regression, and Power disaggregation accuracy (P*_acc_*), as commonly used in NILM [32]. These are calculated as

(15) $$\mathrm{MAE}=\frac{1}{T}\sum_{t=1}^{T}\left|{\hat{y}}_{t}-y_{t}\right|,$$

(16) $$P_{acc}=1-\frac{\sum_{t=1}^{T}\left|{\hat{y}}_{t}-y_{t}\right|}{2\sum_{t=1}^{T}y_{t}},$$

where ${\hat{y}}_{t}$ and $y_{t}$ are the respective decomposition and true values of the target appliance power at time t. Table 1 shows the evaluation metrics for simulations of the ChebNet and AChebNet models.

It should be noted that the disaggregation curves shown in Figure 4, Figure 5, Figure 6, Figure 7 and Figure 8 mainly provide a qualitative comparison of the overall trend consistency and switching-state tracking ability of the models. Such time-domain visualizations are effective for illustrating whether the predicted load follows the main operating pattern of the appliance, but they are relatively insensitive to local point-wise deviations, short-duration spikes, and accumulated small residual errors. By contrast, MAE is calculated over all sampling points and therefore captures these local discrepancies more sensitively. As a result, even when the disaggregation curves of ChebNet and AChebNet appear visually similar in some time intervals, their cumulative absolute errors can still differ substantially. Therefore, the improvement of AChebNet in Table 2 is mainly reflected in its better suppression of local residual fluctuations and point-wise prediction errors, rather than only in large-scale visual differences in the curves.

As shown in Table 1, both ChebNet and AChebNet are capable of effectively disaggregating the power consumption of each appliance, which is also consistent with the qualitative trend observed in Figure 4, Figure 5, Figure 6, Figure 7 and Figure 8. However, although the two models show similar overall trend fitting in some time intervals, AChebNet achieves consistently lower MAE values for all five appliances. This indicates that the advantage of AChebNet is not only reflected in tracking the major operating states of appliances but also in reducing local point-wise deviations and suppressing short-duration residual fluctuations. In particular, the average MAE of AChebNet is reduced by 48.87% compared with ChebNet, and the average Pacc is improved from 87.39% to 92.74%, demonstrating that the proposed adaptive adjacency mechanism improves quantitative disaggregation accuracy even when the qualitative curve differences are not always visually striking.

**Figure 9:**
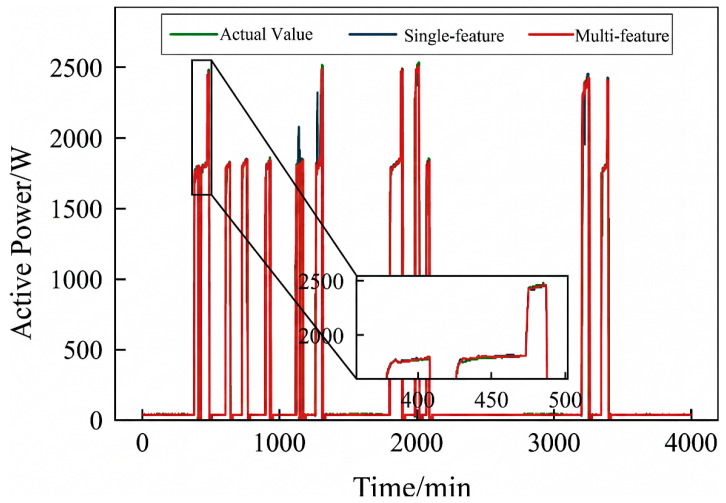
Decomposition effect of an air conditioner under single- and multi-feature input of the AChebNet model.

**Figure 10:**
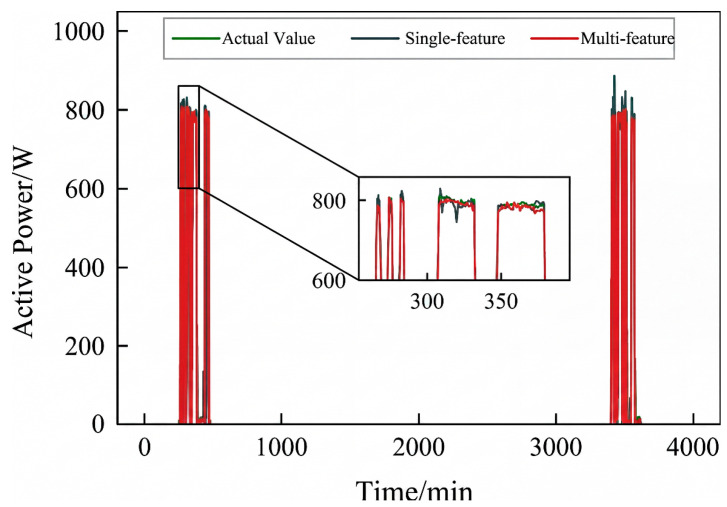
Decomposition effect of a dishwasher under single- and multi-feature input of the AChebNet model.

**Figure 11:**
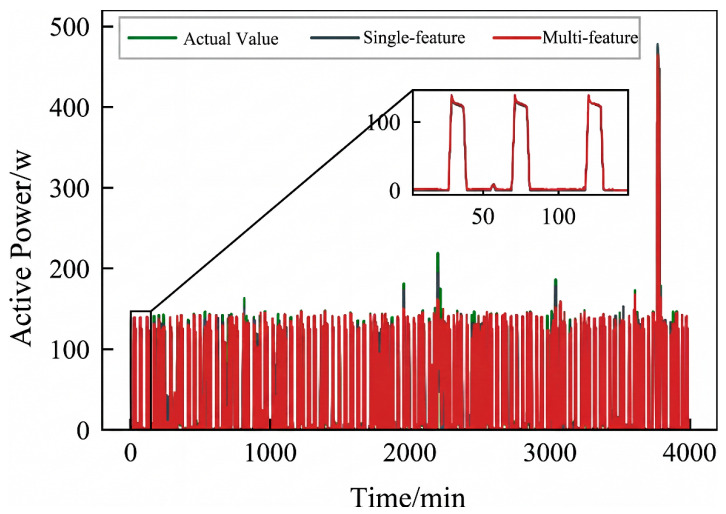
Decomposition effect of a refrigerator under single- and multi-feature input of the AChebNet model.

To analyze the impact of input dimensions on load disaggregation, an input space containing multiple feature elements was constructed by combining four electrical features: steady-state current, active power, reactive power, and apparent power. The proposed AChebNet model was then simulated in single- and multi-feature input scenarios. Figure 7, Figure 8, Figure 9, Figure 10 and Figure 11 show the power disaggregation curves for AChebNet in both cases, and Table 3 shows the comparative results of the load disaggregation metrics.

As can be seen from Figure 9, Figure 10 and Figure 11, the load decomposition effect of the AChebNet model is better with multi-feature input than with single-feature input. For the refrigerator, the load curves obtained under multi-feature input exhibit significantly fewer spikes. For the air conditioner and dishwasher, the operational power disaggregated by the AChebNet model under multi-feature input more accurately tracks the actual operating power. Referring to MAE and Pacc in Table 2, we further compare the load disaggregation performance of AChebNet under single- and multi-feature input. For the five appliances studied, the model’s disaggregation performance is generally superior with multi-feature input, with the average MAE reduced by 30.87%, and the average power disaggregation accuracy improved from 93.22% to 96.17%. The performance improvement for the dishwasher under multi-feature input is particularly notable, with the MAE significantly reduced to 1.13 and Pacc substantially increased to 97.44%. This suggests that enriching the feature dimensions enhances model performance and improves disaggregation accuracy.

### 6.3. Validation and Analysis Based on Measured Dataset

**Figure 12:**
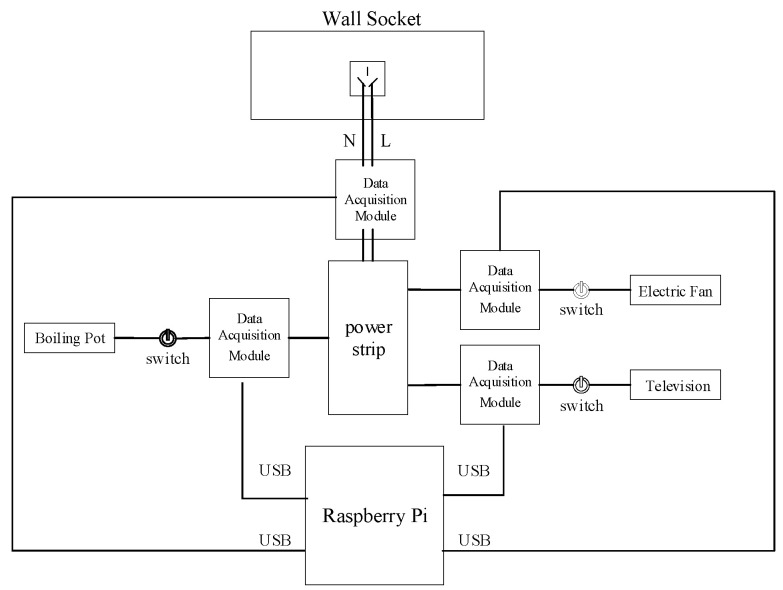
Design diagram of load data acquisition circuit (Raspberry Pi (Raspberry Pi Foundation, Cambridge, UK)).

In this experiment, live and neutral wires were drawn from a wall socket to simulate a residential mains input, creating a small-scale electrical usage scenario. The designed data acquisition circuit is shown in Figure 12. Based on the differences in operating principles of residential electrical devices, the experimental appliances included an electric fan (inductive load), television (power supply load), and boiling pot (resistive load).

**Figure 13:**
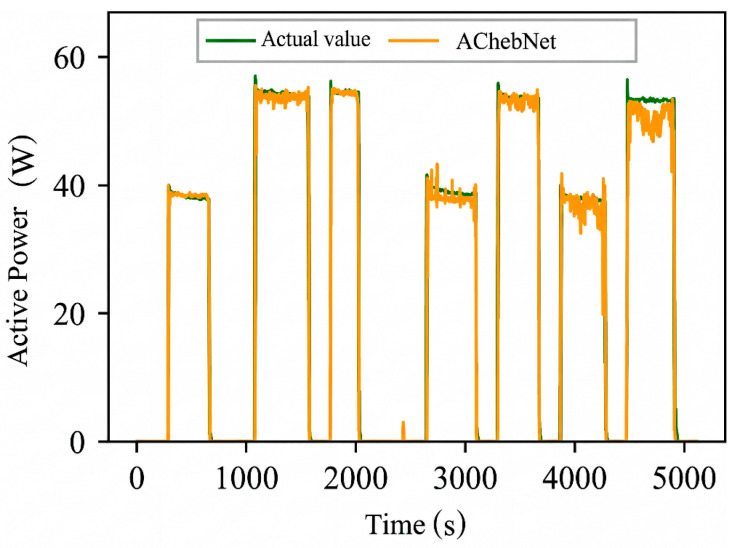
Decomposition effect of an electric fan under the AChebNet model.

**Figure 14:**
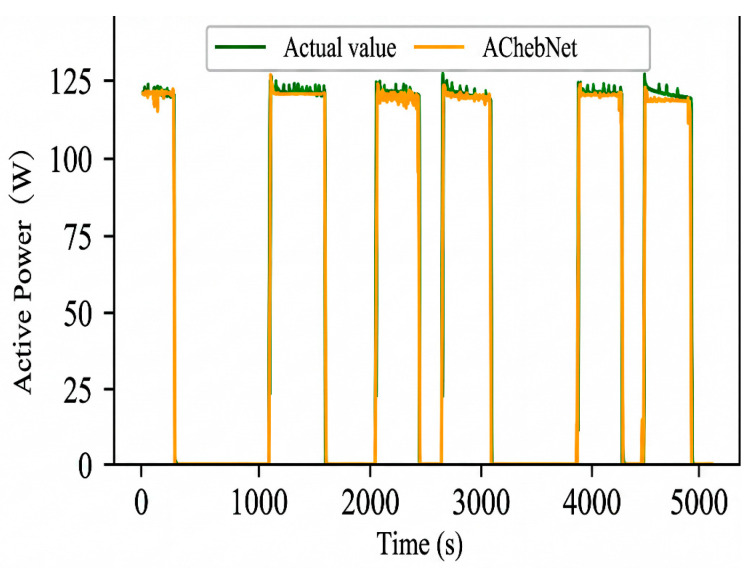
Decomposition effect of a television under the AChebNet model.

**Figure 15:**
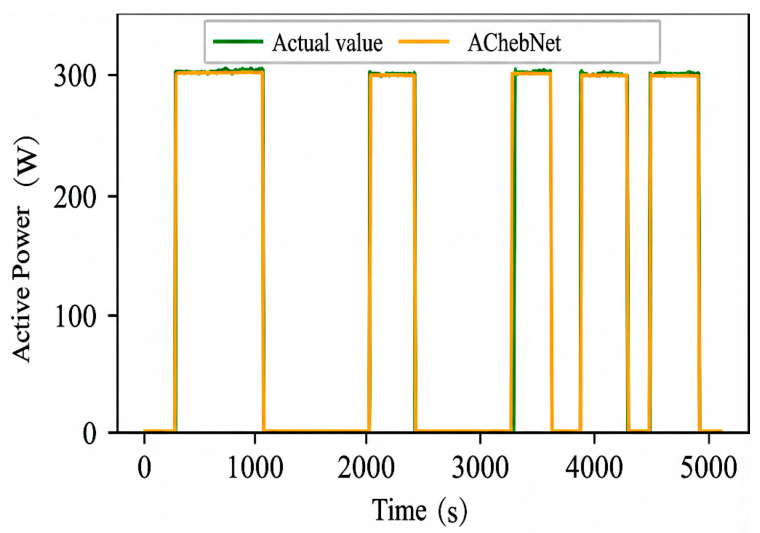
Decomposition effect of an electric boiling pot under the AChebNet model.

The simulation results indicate that the model performs better in load disaggregation with multi-feature input. Therefore, we validated the disaggregation performance of the AChebNet model on the measured dataset with multi-feature input. The measured dataset was independently divided into training, validation, and test sets in a ratio of 6:2:2. The same network architecture and main hyperparameter configuration as those used in the public-dataset experiments were retained for consistency, whereas the model parameters were reoptimized on the measured dataset. In the inference stage, the retrained model was used to estimate the target appliance power for newly acquired sliding-window samples from the same measurement scenario. The results are shown in Figure 13, Figure 14 and Figure 15.

The visualization results indicate that AChebNet demonstrates effective disaggregation performance for all three appliances in the measured dataset. Notably, although the electric fan’s disaggregation performance is slightly less than that of the other two appliances due to its two power states and complex operating conditions, AChebNet can still accurately capture its on-off transitions. For the television, a switching power supply load with current transients, the model also achieves a good fit. For the other two appliances with single operating states, AChebNet accurately tracks load switching events and successfully and precisely disaggregates the power consumption values.

While the experiments on the AMPds2 public dataset were mainly designed to verify the effectiveness of the proposed adaptive adjacency matrix and the contribution of multi-feature input, the measured dataset was further used to evaluate the practical applicability of AChebNet under a real measurement scenario. To this end, AChebNet was compared with the seq2point model [33], the FHMM from the NILMTK toolkit, and three recent representative NILM models, namely STNILM, NILMFormer, and MAMBA2NILM, based on MAE and Pacc. The comparison results are shown in Table 4.

Table 3 shows that AChebNet outperforms all comparison models, including both classical baselines and recent NILM benchmark methods, with respect to MAE and Pacc. Specifically, the proposed model achieves the lowest mean MAE of 0.78 W and the highest mean Pacc of 99.22% across the three real-world appliances. Compared with the recent models STNILM, NILMFormer, and MAMBA2NILM, AChebNet still maintains superior decomposition performance. Compared with the classical seq2point and FHMMs, the improvement is more pronounced, as the mean MAE is reduced from 6.79 W and 16.49 W to 0.78 W, while the mean Pacc is improved from 91.24% and 84.50% to 99.22%, respectively. This indicates that the proposed adaptive graph convolution mechanism is more effective in modeling both temporal relationships and latent feature correlations in measured load data.

### 6.4. Ablation Study of Key Components

To further validate the individual contributions of the adaptive adjacency matrix and multi-feature fusion, ablation experiments were conducted under a unified setting, as shown in Table 5. Model M1 denotes the baseline ChebNet with single-feature input. On this basis, M2 introduces multi-feature fusion only, M3 introduces the adaptive adjacency matrix only, and M4 represents the complete model with both components retained.

The results show that both modules contribute positively to the final performance. Compared with M1, M2 reduces the MAE from 13.69 W to 11.42 W and improves Pacc from 87.39% to 89.85%, indicating that multi-feature fusion enhances the descriptive ability of node attributes. Compared with M1, M3 achieves a more significant improvement, with MAE reduced to 7.00 W and Pacc increased to 92.74%, demonstrating the effectiveness of the adaptive adjacency matrix in capturing latent inter-node dependencies. When both modules are simultaneously adopted, M4 achieves the best performance, with MAE further reduced to 5.21 W and Pacc improved to 94.58%. This indicates that the two components are complementary and jointly improve load disaggregation accuracy.

## 7. Conclusions

Most nonintrusive load disaggregation models focus primarily on the sequential input of data in Euclidean space and single electrical features. The correlations between appliance operating states have not been sufficiently explored. We proposed a nonintrusive load disaggregation model grounded in spectral graph theory and based on adaptive graph convolutional neural networks. The principle contributions of this study are summarized as follows.

The model was constructed based on spectral graph theory to build data input, combining the ChebNet model with the proposed adaptive matrix to perform feature extraction and information aggregation, successfully realizing high-precision noninvasive load decomposition.

We discussed the impact of input dimensions on the disaggregation results of the AChebNet model, which demonstrated superior disaggregation performance under multi-feature input compared to single-feature input.

Connecting the proposed model with multi-feature inputs produced better results than mainstream networks, with an accuracy of 94.58%.

## Figures and Tables

**Table 1:** Training hyperparameter settings of the proposed AChebNet model.

Hyperparameter	Setting
Optimize	Adam
Loss function	MSE
Initial learning rate	0.001
Weight decay (L2 regularization)	1 × 10^−4^
Batch size	64
Number of epochs	100
Learning rate decay	Reduce by 0.5 every 20 epochs

**Table 2:** Evaluation indices of ChebNet and AChebNet.

Electrical Appliances	ChebNet		AChebNet	
	MAE (W)	P*_acc_*(%)	MAE (W)	P*_acc_*(%)
Lamp	12.96	89.87%	8.55	93.32%
Air conditioner	14.93	96.97%	4.66	99.05%
Dishwasher	6.27	85.83%	4.72	89.33%
Refrigerator	25.38	73.90%	8.49	91.27%
Television	8.89	90.37%	8.56	90.73%
Mean	13.69	87.39%	7.00	92.74%

**Table 3:** AChebNet model input load decomposition index results in single- and multi-feature cases.

Electrical Appliance	MAE (W)		P*_acc_*(%)	
	Single	Multi	Single	Multi
Air conditioner	4.66	3.19	99.05%	99.35%
Dishwasher	4.72	1.13	89.33%	97.44%
Refrigerator	8.49	8.04	91.27%	91.73%
Mean	5.96	4.12	93.22%	96.17%

**Table 4:** Load decomposition evaluation indicators for six Models.

Index	Model	Electric Fan	Television	Boiling Pot	Mean
MAE (W)	AChebNet	0.69	0.88	0.76	0.78
	MAMBA2NILM	1.21	1.34	0.91	1.15
	NILMFormer	1.74	1.56	0.98	1.43
	STNILM	2.31	1.95	1.18	1.81
	seq2point	8.47	9.39	2.52	6.79
	FHMM	12.77	11.08	25.62	16.49
P*_acc_* (%)	AChebNet	98.67%	99.25%	99.73%	99.22%
	MAMBA2NILM	97.86%	98.64%	99.48%	98.66%
	NILMFormer	97.08%	98.31%	99.41%	98.27%
	STNILM	96.12%	97.85%	99.26%	97.74%
	seq2point	82.63%	92.12%	98.97%	91.24%
	FHMM	73.45%	90.54%	89.50%	84.50%

**Table 5:** Ablation experiment results of key components.

Model	Adaptive Adjacency Matrix	Multi-Feature Fusion	MAE (W)	Pacc (%)
M1	×	×	13.69	87.39
M2	×	√	11.42	89.85
M3	√	×	7.00	92.74
M4	√	√	5.21	94.58

## Data Availability

The data and code are considered intellectual property of the project and are therefore not publicly available.
